# Exploring the lived experiences of early- and mid-career female academic GPs in the UK: a biographical narrative interview study

**DOI:** 10.3399/BJGP.2025.0359

**Published:** 2025-11-04

**Authors:** Ellen MacIver, Pavel Ovseiko, Eleanor Barry, Trisha Greenhalgh

**Affiliations:** 1 Nuffield Department of Primary Care Health Sciences, University of Oxford, Oxford, UK; 2 Radcliffe Department of Medicine, University of Oxford, John Radcliffe Hospital, Headington, Oxford, UK

**Keywords:** academia, general practice, qualitative research, women

## Abstract

**Background:**

Women are underrepresented within academic general practice, particularly after mid-career.

**Aim:**

To explore the lived experiences of early- and mid-career female academic GPs and inform ways to reduce attrition through the GP academic career path.

**Design and setting:**

This was an in-depth qualitative interview study within the UK.

**Method:**

Adapted biographical narrative interpretive method interviews were utilised, analysed thematically using Braun and Clarke’s reflexive method, and informed by Bourdieu’s theory of practice. Composite narratives were developed as part of the analysis to identify key biographical storylines and to present findings.

**Results:**

In total, 39 interviews with a diverse sample of 13 female academic GPs were conducted. Five composite narratives reflecting corresponding themes were generated. Participants described: challenges in ‘thriving? or surviving?’ in academic general practice; ‘feeling on the cliff edge’ with precarious careers balanced against fulfilment and creativity; the cumulative burdens of ‘doing the juggle’; and living between ‘two worlds’. Women who followed conventional academic career pathways appeared more positive within their careers than those who did not. Women who entered later in their GP careers and those who experienced multiple forms of disadvantage reported additional barriers. Participants described their efforts managing practical and ethical tensions between their clinical, academic, and personal responsibilities.

**Conclusion:**

Women academic GPs live complex and demanding lives. Different strands of their unfolding life narratives — as clinicians, academics, and partners and/or carers — generate recurrent tensions and conflicting pressures. Experiences are varied. Academic support structures should address (among other things) the career-limiting impact of short-term contracts, mentorship, and inequity in navigating the field.

## How this fits in

This is the first study, to our knowledge, to explore the lived experiences of female academic GPs in combining clinical, academic, and other commitments. Concerningly, there is a drop in women remaining within primary care academia and progressing to higher roles such as leadership and professorship. Gender parity throughout all stages is vital to ensure primary care research is relevant and inclusive. Academic GP careers have gained little focus in research so far, this study builds on this deficit to provide deeper understanding of women’s experiences as clinical academic GPs and guidance for potential support strategies.

## Background

Women currently make up the majority of the GP workforce (58%),^1^ yet there remains substantial gender inequity within academic medicine, particularly in senior roles.^2,3^ High attrition rates in mid-career^4–6^ have been termed the ‘leaky pipeline’.^7,8^ As of 2023, only 34% of academic general practice professors were women.^3^ Evidence from within clinical and academic medicine suggests that women remain undervalued,^2,9–11^ adversely affected in their careers by both direct and indirect sexism, and by institutional, individual, and societal factors.^2,4,5,11–13^ Barriers to progression and exit points in academic pathways have been previously explored;^12–15^ however, women’s careers in academic general practice remains underresearched.

In the UK, there are a variety of routes for GPs into academia.^16^ All GPs undertake a 3- to 4-year GP specialty training programme. Some apply for academic roles during training, via schemes such as the National Institute for Health and Care Research (NIHR) academic clinical fellowship.^17^ Others enter via alternative pathways including medical education, schemes for mid-career entry, or through individualised routes.^15,18^

This study is the first, to our knowledge, to examine the lived experiences of early- and mid-career female academic GPs and was conducted as part of an NIHR-funded in-practice fellowship for the first author. The study was framed using an interpretivist approach, with the intention to elicit the ‘lived, experienced and narrated’ life with a phenomenological lens, focusing on the experiences as interpreted and understood by the participants.^19,20^

The objectives of the study were to:

- develop a deeper understanding of the experiences of early- and mid-career women academic GPs in balancing academic, clinical, and other commitments;
- explore the lived experiences of this group, with a focus on key areas of attrition; and
- explore the institutional barriers faced by female academic GPs in their career choices/progression and ascertain support structures or organisational factors that are required to increase retention.

## Method

We conducted an in-depth qualitative interview study using an adapted Wengraf’s biographical narrative interview method (BNIM) to elicit lived experiences and perceptions.^19,21^ The findings have been reported in line with the COnsolidated criteria for REporting Qualitative research (COREQ) checklist (Supplementary Table S1).^22^

### Study context and governance

This study was designed using a constructivist epistemological perspective, recognising the role of interpretation and social construction in knowledge creation. The lead researcher (the first author) is a GP and NIHR in-practice fellow with academic interests in gender inequity within the medical profession. Reflexivity was conducted throughout, including reflexive journalling and challenging preconceptions. Braun and Clarke’s reflexive thematic analysis recognises that bias cannot be eliminated, but requires recognition, acknowledging the researcher’s role in generating data.^23,24^

### Sampling and recruitment

Participants were invited to participate via academic general practice departmental mailing lists and wider professional networks. Snowball and purposive sampling were used. Participants were selected for a maximum-variation sample of female GPs from diverse backgrounds, ranging in age, general practice and academic job roles including time served, location, and caring responsibilities.

All participants were required to be female, qualified GPs (post-Certificate of Completion of Training), employed (or previously employed) in an academic general practice role.

### Data collection

In total, 39 interviews were conducted by the first author. An adapted BNIM framework drawing on that employed by Macfarlane *et al* ^25^ was used. Interviews lasted between 45 and 70 minutes. BNIM restricts interviewer interventions initially to the single question used to induce narrative (SQUIN). The SQUIN was developed to focus participants' narratives on their individual career stories and events they perceived to be important; further detail is provided in Supplementary Table S1. Following the SQUIN, the interviewer is then restricted to encourage the narrative, without asking new questions.^19,21^ A second interview based on the narrative was conducted, then a final semi-structured interview after initial analysis.^19,26^ Depending on duration of the initial narrative, most interviews were conducted in three separate sittings; for some the first and second were consecutive. Of the interviews, 35 were conducted online via Microsoft Teams and four conducted face-to-face as per participant choice. Interviews were audio-recorded and transcribed using Microsoft Teams transcription functionality. Recordings were listened to and transcripts edited to ensure verbatim accuracy by the first author. Field notes and reflective entries were included in the dataset.

### Data analysis

Data analysis occurred in several stages: immersion, development of inductive codes and themes, narrative illustration, and validation. The first author read and re-read transcripts alongside listening to the audio-recordings to gain familiarity. Transcripts were coded manually with provisional handwritten codes, then transferred to NVivo 14 for development. The three interviews for each participant were analysed for narrative features and individual storylines, then the dataset in its entirety was reviewed for common themes, noting areas of silence or disagreement. Themes were built on and developed iteratively with constant comparison with the data^23,24^ by the first author with support from the senior author and the second author, and with reference to Bourdieu’s theory of practice.^27–29^

### Composite narratives

Composite narratives involve the development of a ‘shared’ narrative, incorporating findings from multiple participants, into a unifying voice, and have been used in similar qualitative research.^30–39^ Composite narratives were adopted to situate the findings of the data within their context,^40^ and to encapsulate the complexity of experiences, used as both an integral component of the analysis and to present our findings.^41^ The narratives were developed as outlined by Johnston in the six-step process for composite narrative development,^42^ through integrating insightful quotes and interviews pertinent to a theme then distilling this into a coherent narrative.^43,44^ The content was derived from verbatim quotes and minimally fictionalised to ensure anonymity.^39^ In the validation stage, the narratives were refined by the first author, second author, and senior author. Any opinions or expressions were taken directly from participants, and the finalised narratives crosschecked with participants for anonymity and validation.

### Theoretical framework

Bourdieu’s theory of practice was utilised as a sensitising lens for the analysis in development of inductive codes and subsequent themes (Supplementary Box S1).^27–29,45^ Practice relates to how individual actions, societal structure, and meaning is given to the sphere of academic general practice as a social space where interactions, negotiations, and internal power structures compete. Individuals compete within this field using expected norms, power, and invisible rules.^45,46^ Agents have varying levels of power in the forms of capital (acquired forms of power) and habitus (individual dispositions that guide one’s interactions and behaviour), which interact ‘within a field to produce a profession’.^29,45^ Adopting this framework when developing our themes enabled us to focus on both individual and societal influences on women’s careers. Through exploring habitus — how women’s perceptions of themselves were constructed, their experiences of entry into academic general practice, and the influence of symbolic capital in the forms of social (such as networks, support structures, family) and cultural capital (qualifications, education) — additional meaning was given to the women’s experiences with critical reflection on unwritten norms and power struggles.

## Results

Overall, 13 female academic GPs were recruited. Basic demographic details are included in Table 1. The length of time within academia ranged from 2 to 18 years, in clinical practice from 2 to 25 years. All self-identified as early- and mid-career academic GPs. No participants opted to leave the study.

**Table 1.** Self-reported characteristics of participating female academic GPs (*N* = 13)

*Characteristic*	*N*	%
**Age, years**		
30–39	7	54
40–49	5	38
>50	1	8
**GP role**		
Partner	2	15
Salaried	6	46
Retainer	3	23
Locum	1	8
OOH	1	8
**Academic role**		
Pre-doctoral	3	23
Doctoral student	3	23
Post-doctoral	4	31
Teaching fellow	2	15
Previous academic trainee	1	8
**Time within clinical practice post-CCT (years)**		
<5 years	3	23
5–10 years	6	46
>10 years	4	31
**Time within academia (years)**		
<5 years	2	15
5–10 years	7	54
>10 years	4	31
**Additional caring responsibilities**		
Children	10	77
Older relatives	1	8
None	2	15
**Ethnicity**		
White British	6	46
White European	1	8
Minority ethnic groups	6	46

CCT = Certificate of Completion of Training. OOH = out-of-hours service.

The findings are presented within the corresponding themes and composite narratives (Boxes 1–5; text in italics are direct verbatim quotes from the participants), to give a rich, unified voice to the participants in the form of anonymised shared narratives. The themes are summarised with further illustrative pseudonymised quotes in Supplementary Box S1.

Box 1. **Tanvi, a composite narrative on the theme — challenges of thriving and surviving**

*If you weren't in the top ten per cent, or if you weren't sort of publishing whilst being a medical student, then it was sort of perceived that research wasn't for you. I'd never considered the option of being an academic GP because I'd never really seen what that looked like.* So, I worked as a GP for about ten years but had all these ideas. I contacted my local department, they suggested I apply for some funding. But when I looked at the schemes I didn’t *really fit in the box. Like you know, I'm older, different experience … like what does the system do with me?* I really struggled to know what to do, *I was thinking how do I move sideways? Is there a way? Am I too old? I don't have any skills. Basically, I've got nothing to offer and there isn't a route for people like me.* It sometimes feels like you must be the *right type of person to fit in.* Luckily, I made a great connection in the department, and *you need to have a mentor who has unbelievable faith in you.* Meeting mine was just a *pivotal moment in my career* but it happened *by chance. She’s just an incredible woman … she’s like high challenge, high support.*

Box 2. **Helena, a composite narrative on the theme — feeling on the cliff edge**

I’ve had to sacrifice my own time to keep up, *one of the negative things I think maybe about academia ... it's kind of basically never ending … it's kind of hard to draw the line where to stop.* It’s also incredibly stressful, *the pressure of academia … you've got to publish or perish. Get papers out. Make sure you get another grant. I've got to keep up with everyone. If I don't do this, I'm going to fall off a cliff. I’ve also had to self-fund further qualifications, and I'm being paid less, but taking on more responsibility and I'm more qualified now.* Having these short-term contracts are tough because it's just really difficult to plan anything … *because I might be applying for a job or I might have just started a new job.* I’m not sure now whether I really want to be a professor, whether *getting to the top of the ladder and having the title isn't necessarily the be all and end all. I might be in with a chance, but then I'm asking myself, do I actually want it? And actually, the more I ask myself that, the more I'm thinking. No.*

Box 3. **Gillian, a composite narrative on the theme of ‘It’s wonderful chaos’**

When I started my GP training *I was immediately fascinated by the one [the patient], the people you know and talking to and getting to know people … and the depth of the kind of knowledge that was needed was fascinating* but I think I always felt there was a *part of me I was not serving,* that I needed something more creative. *In fact, and actually looking back, I think I've always found it difficult being a clinician … without having a space to be creative and use my brain in a different way ... For me doing clinical academic work is a way for me to maintain my creativity and my interests.*

It’s also great to think about the positive impact my academic work is having, *this is stuff that has potential to change lives* which is just amazing. I *genuinely am passionate about it, not because it’s putting me on a trajectory or a career path that will get me a lot of money or success. It’s actually not about that, at all. It’s just because I love what I do.*

Box 4. **Aditi, a composite narrative on the theme — ‘doing the juggle’**

I assumed I would continue the same trajectory after having my kids. Although my academic department and GP practice are supportive, it’s been tough, it’s just *madness basically.* Especially on my GP days, *I am in at eight and often don’t leave until seven, but the nursery shuts at six. I have my husband who’s really supportive. He’s not a doctor, which actually I think is very, very helpful because he doesn’t do like nights or weekends. He just like does a nine to five and then works from home a lot. So actually like the little practical things, make a huge difference, I think when you’re a clinician. Although we are fifty:fifty … I’ve taken on the mental load of the kids. I run the household. Luckily, my partner is not clinical so he kind of picks up the pieces for me if something were to happen … there’s, you know, certain immovability of clinical practice sometimes, I can’t tell you when I’m going to be back and so you’re going to have to be the point person [the primary contact for certain responsibilities].* The academic work is much more flexible which has *enabled me to be there more for my kids,* but it’s a balance as I feel I’m working both jobs more than part-time and then there’s little time left for anything else.

Box 5. **Fiona, a composite narrative on the theme — living between two worlds**

I think *having people like myself who understand both sides of the coin are really important,* but it’s hard being essentially *in-between worlds, and you’re constantly context switching.* Overall, having both jobs *has really enhanced how able I feel to do my clinical job and how much I enjoy it … there's the clinical value that you bring to academia and the academic value that brings to clinical, I feel like that was an untapped space. I think having this dual career for me is a much more sustainable option than being a long-term full-time NHS GP.* Having both roles, *it has lots of challenges but different challenges, so they sort of balance out somehow … it does mean more balls to juggle, and especially when there's other stuff at home that can be challenging at times.* I do also feel guilty, *I'm twenty-five appointments down because I'm not working an extra day, that's that made me feel guilty.* The job has changed a lot though; *I mean general practice is a bit of a mess at the moment.* It’s a worry as well, clinical was my *plan B, but now there's all this stuff about those ARRS [Additional Roles Reimbursement Scheme] roles … there's not really a need for any locums … so pretty grateful, really, that I've got this PhD to fund most of my time.*

### Surviving? Or thriving?

There was variation between participants, with some ultimately thriving within academia and others facing challenges simply to survive. This often corresponded to accrued levels of cultural and social capital held within the field of academic general practice, reflected in Tanvi’s narrative in Box 1.

#### Being the ‘right type of person’

There was a perception that to gain entry and to thrive within academic general practice you had to be ‘the right type of person’*.* Not being from a middle-class, doctor-parent background was felt by some to be a disadvantage, particularly in obtaining tacit knowledge and pre-existing networks to facilitate entry (see Supplementary Box S1).^47^ The feeling of not being ‘the right type’ delayed entry for some and led to feelings of imposter syndrome. For example, one participant expressed that her appearance and how she spoke ‘marked*’* her. Several talked of being ‘found out’ and expressed surprise at securing academic roles. This perception aligns with Bourdieu’s habitus, in which one’s internalised dispositions reflect that of a particular social group, and affect how one would respond or interact in certain contexts, through their habits, skills, perceptions, and thoughts.^27–29^

Entry to the specialty was challenging for those who held less cultural and social capital, and this was particularly true for those who entered outside of academic training pathways (Box 1). These women described challenges obtaining opportunities they saw others achieve, sensing that their habitus and the field were ‘mis-matched’.^27–29,48^ Economic capital was also pertinent in that some could afford to fund additional qualifications or access entry via unpaid roles, which resulted in greater symbolic capital and higher positionality within the field.

A lack of representation was noted, for instance, on funding panels and grant boards, and the women from minority ethnic groups reported that this could be alienating. Although many recognised there did seem to be a drive towards increased diversity through schemes such as Athena SWAN charter and greater recognition of intersectionality in academic careers.

#### Support

Support from influential mentors was felt to be critical as both role models and in increasing the women’s social capital, creating networks and opportunities. These relationships usually developed organically over time and were notably more established for those who had undergone academic training. Those who struggled to secure these relationships presented their career paths as more turbulent.

### Feeling on the cliff edge

Helena’s narrative (Box 2) illustrates the tensions in combining clinical and academic work, alongside additional responsibilities, with the insecure career structure. Many reported experiencing fear and anxiety, that they may ‘fall off the cliff’ at any moment.

#### Living in the uncertainty

The precarity of academic careers was a persistent thread and the impact of short-term contracts was a concern. For the women with longer funding, such as PhDs, the anxiety was felt less acutely than by those facing the end of funding. The women aiming to secure highly competitive post-doctoral grants reported feeling particularly anxious. Holding higher levels of cultural and social capital appeared to be influential in how secure the women felt ‘living in the uncertainty’ and thus their position within the field. For example, those who had followed linear progression were often more assured, and perceived themselves to operate within the field well, whereas those who struggled spoke of anxiety and stress with each career stage.

#### Getting the balance right

Academic careers were seen as insecure, and many discussed their career planning with trepidation (Box 2). Some expressed concerns regarding retention of women to higher grades because of inadequate career support and the personal and financial sacrifices that were perceived necessary to succeed. For those nearing more senior roles, many questioned whether it was ‘actually worth it’. Tensions were often felt between developing as an academic and meeting demands of family and caring responsibilities. Some found it challenging to present themselves as expected for promotion. This was particularly prevalent in the narratives of women from Asian backgrounds, who spoke of an ingrained sense of duty to put others above themselves, demonstrating the influence of their individual habitus on their interactions with the academic field.

### ‘It’s wonderful chaos’

Despite the challenges, the experience of academic work was principally positive with the women expressing fulfilment and passion, as illustrated in Gillian’s narrative (Box 3).

#### Academia as creative outlet

Initial scene setting for many of the narratives placed emphasis on the woman’s character. Many described themselves as having a creative side, which clinical medicine alone did not serve. Creativity was strongly valued, borne out of their earlier life experiences, enmeshed within their habitus. The narratives included feelings of being stifled by inflexible clinical training. Academic general practice in contrast valued creativity, critical thinking, and intellectual freedom outside of, and complementary to, clinical work.

#### Fulfilment and successes in academic general practice

Observing the tangible impact of their research or roles within education on clinical practice and policy was immensely rewarding. Accruing cultural capital in the form of successes (such as securing large grants) led to the women operating with increasing confidence, thus influencing their evolving habitus and interactions with the field itself. For most, these benefits often counteracted the challenges that came with the career pathway, although one participant ultimately left academia because of the associated job insecurity compared with GP partnership.

### ‘Doing the juggle’

Aditi’s narrative (Box 4) summarises the challenges of balancing additional responsibilities such as being a carer or having children, which was felt acutely by many of the women.

#### Navigating the three separate spheres of general practice/academia/home

The cumulative impact of the three separate spheres of general practice/academia/home often meant limited personal time and all perceived that they ended up working more to keep up. This ‘triple role’ often resulted in overwhelming accumulation of outcompeting demands. The continued struggle between prioritising family or home life and achieving academic expectations was at the forefront of many of the narratives; however, academia conferred increased flexibility, compared with clinical work, which was highly valued.

#### Experiencing motherhood as an academic GP

For those who had children, the transition into becoming a clinician–mother was a significant turning point and, for most, this transition featured prominently. Being a GP, although traditionally viewed as more family friendly, posed certain challenges with inflexible clinics. Many relied on family members and partners to be the ‘reliable one’ there for pick-ups and drop-offs, as illustrated by Aditi (Box 4). Despite often splitting responsibilities for childcare and domestic work, many felt the overall mental burden of managing the family, along with persisting societal gendered expectations surrounding hidden work, lay largely with them.

In the narratives of the women who had children, all reported their career progression to be slower compared with (often male) colleagues, because of part-time working, being less flexible owing to additional responsibilities, and the implicit (often out-of-hours) work required to succeed. This ‘maternal wall bias’ was balanced against the fulfilment from their family lives, and many expressed acceptance of their differing career trajectories. For those planning to have children, the insecure academic career structure was felt to be at odds with reproductive planning.

### Living between two worlds

Fiona’s narrative (Box 5) summarises the unique experience women described operating within the two worlds of clinical general practice and academia.

#### Synergy

Being able to bring a clinical perspective to the academic was felt to be highly valuable, enabling the women to adopt unique roles within interdisciplinary teams. Having both roles was felt to confer increased sustainability and, for some, respite from the demands of clinical practice.

Those who had reduced clinical work reported feeling guilty and expressed concerns about how they were perceived by colleagues. This was prominent in the narratives of the women who had been in their practices for long periods or who entered academia later in their career, compared with those who had followed academic training pathways.

#### Changing landscape of general practice

The context of UK general practice was a concern. All felt that the GP role had changed substantially and that clinical pressures were unsustainable. There were increasing tensions within the narratives. Many felt reassured by their academic role, but experienced anxiety about the lack of clinical work available (because of the current GP unemployment crisis) should they become un- or underemployed. This was particularly pertinent in the narratives of women who were facing the end of funding.

### Analytical synthesis

Bourdieu’s theory of practice allows us to consider the experiences of the women beyond the individual, to gain a deeper awareness of the wider influences on women’s academic careers and successes. The women operated within academic general practice not solely as individuals, but with interwoven, competing influences. Academic general practice as a field provoked unique challenges through the uncertain competitive nature and competing demands of academic and clinical spaces. For Bourdieu, habitus is *‘embodied history, internalised as a second nature and so forgotten as history’*.^28^ The individual habitus was seen to influence reasons for entering the field and subsequent interactions within academic general practice. For all women they spoke of passion for academic work, often stemming from prior academic experiences, a desire to influence clinical practice, and for some ingrained aspirations from childhood.

The relationship between habitus, academic general practice as a field, and varying levels of social and cultural capital held by the women was seen in the narratives to operate at multiple levels. Symbolic capital — namely social and cultural capital — led to inequity in experiences, opportunities, and, ultimately, career progression. As highlighted in Tanvi’s narrative (Box 1), this was particularly pertinent for the women entering academia from the outside. The influence of the wider sociocultural context of the women also had an impact on their careers. Those with greater accumulation of social capital, for instance those with supportive mentors and networks, ultimately thrived more successfully. Critically, those who struggled were often experiencing multiple competing streams of disadvantage. For example, one participant recounted the challenges she faced becoming established in academia, when she was also a carer and lacked access to essential resources. Those with supportive family members or spouses were able to be more flexible with their work, or to work more unsociable hours and meet academic demands.

## Discussion

### Summary

In this study we have explored how a diverse sample of female academic GPs experience their clinical academic careers alongside other responsibilities. GP academic career pathways are varied, ranging from entry via academic training programmes to later in a career, through formalised schemes or individualised routes. Many entry routes require a certain level of prior achievement or knowledge to be successful. Women entering in mid-career, particularly, felt these routes were exclusionary and required additional personal and financial sacrifice to succeed. The influence of habitus, cultural, social, and financial capital on academic career progression was reflected in women’s narratives, and the women had varied experiences of negotiating entry and thriving within the field. Those who were most successful were supported by influential mentors and social support networks, had the ability to access additional resources, and whose habitus ‘fit’ with the field.^27,28^ Our analysis suggests that the women who experienced additional disadvantages, for example, those from poorer socioeconomic backgrounds or who were single parents and solo carers for relatives, reported more challenges in accruing capital and operating within the field.

Being a female academic GP involves significant challenges, often having an impact on work–life balance, increased job insecurity, personal and financial sacrifices, and additional stresses combining clinical and academic work. However, academic general practice provides creativity, flexibility, respite from clinical demands, and a chance to carve a unique role within teams, improving professional satisfaction.

The repercussions of unclear career pathways, particularly short-term contracts, on long-term career intentions warrants addressing to reduce attrition rates. Recognition of the challenges that women who are experiencing intersectional barriers face while navigating academic general practice needs to be addressed.

### Strengths and limitations

This is the first study, to our knowledge, that explores the lived experiences of this group, adding insights into academic general practice and women’s careers. The BNIM generated rich, in-depth qualitative findings and the composite narratives have presented these in a unique, accessible format.

One weakness is that women may have volunteered to participate because of adverse experiences and therefore the sample may be self-selecting. The participants were all based in departments in England and experiences within the devolved nations may differ. There was only one participant who had left academic general practice within the sample. To understand attrition experiences, further research with this cohort is needed.

### Comparison with existing literature

The findings are largely consistent with relevant literature relating to academic careers;^13–15,49,50^ however, provide new insights into academic general practice and attrition experienced specifically by women. Similarly to McElhinney and colleagues’ studies exploring academic general practice careers within an education department^30^ and GPs in Scotland,^15^ our study has found that pursuing a career in academic general practice poses significant challenges. Nonetheless, once within the field, experiences are largely positive. Academic general practice provides creativity, fulfilment, and enrichment, which aligns with other studies.^13,15,30,51,52^ Influential mentors and trailblazing supervisors were seen to be pivotal in ensuring career success, and the importance of mentorship has been widely reported in other studies related to academic clinical careers.^13,15,30,49,51–58^ However, this study highlights that certain women are struggling to access these relationships and are potentially being disadvantaged in their career progression as a result. The lack of women in higher leadership roles and as mentors in academia is troubling,^4,5,13,52,55,59,60^ and our findings support that there is limited enthusiasm from early- and mid-career women to seek higher roles.

Critically, this study illustrates that women negotiating entry into academic general practice from outside (usually in mid-career) face increasing barriers. The Walport report,^61^ and subsequent reforms, established structured pathways into academia, and the women who had followed training routes reported fewer barriers. Entry to, and succeeding in, academic general practice later required personal financial sacrifice and lengthy perseverance, which is reflected in the literature related to clinical academic careers.^12,45,62^ Unclear career pathways have been previously established to have an impact particularly on women and those in early careers. This study highlights that this remains a concern despite previous calls for reform.^4,11,55,62^

The wider literature highlights experiences of direct discrimination because of the ‘maternal wall bias’,^4,5,8,11,13,50,59,63,64^ as well as the effects of persistent societal gender roles on hidden work and domestic responsibilities.^65–69^ Reports of targeted discrimination such as direct loss of opportunities were lacking within the empirical data. However, women reported slower career progression, challenges balancing the ‘triple role’, and increased mental burden associated with societal expectations of traditional gender roles.

### Implications for research and practice

We recommend a taskforce is developed by the Royal College of General Practitioners in discussion with other interested stakeholders, such as the National Institute for Health and Care Research, the Society for Academic Primary Care, and the British Medical Association’s Women in Academic Medicine Committee, to examine the underrepresentation of women in academic general practice at higher levels, and the barriers outlined, focusing on the following areas:

- inequities between those who enter via training routes and those who enter later and/or via less traditional routes;
- a specific pathway that enables established female GPs to gain experience and access academic networks and support, before applying for further funding, to address the imbalance;
- support structures, mentoring, and networking within, and between, departments;
- intersectional barriers to accessing support, opportunities, and progression;
- the precarity of academic general practice and the instability of short-term contracts;
- improved access to bridging funding for those with additional responsibilities; and
- re-evaluation of the metrics required for progression.

Further research could usefully explore additional barriers in women who have been unsuccessful in their attempts to secure academic general practice careers, as well as insights from women who have left.

In conclusion, this study demonstrates that women’s careers in academic general practice are highly varied, although academia brings additional enrichment and sustainability overall. There appears to be inequity between women who enter via training routes and those who enter later in their careers. Drawing on Bourdieu’s theory of practice has shed light on this phenomenon. Those who enter via training routes accrue greater social and cultural capital, which aligns their habitus more closely with academic general practice, than those who do not. Women who enter later face additional burdens, often financial and personal, to thrive within the academic space. All women in academic general practice face additional challenges balancing demanding, and often competing, dual careers with additional responsibilities. To reduce attrition rates, the mental burdens of the ‘triple role’ need to be addressed through improved awareness, as well as adequate mentorship, support, and career planning at all stages. The career-limiting impact of the unstable career path, particularly short-term contracts, needs urgent re-evaluation.
